# Multi-level high utility-itemset hiding

**DOI:** 10.1371/journal.pone.0317427

**Published:** 2025-02-03

**Authors:** Loan T. T. Nguyen, Hoa Duong, An Mai, Bay Vo

**Affiliations:** 1 School of Computer Science and Engineering, International University, Ho Chi Minh City, Vietnam; 2 Vietnam National University, Ho Chi Minh City, Vietnam; 3 Faculty of Information Technology, HUTECH University, Ho Chi Minh City, Vietnam; Sejong University, KOREA, REPUBLIC OF

## Abstract

Privacy is as a critical issue in the age of data. Organizations and corporations who publicly share their data always have a major concern that their sensitive information may be leaked or extracted by rivals or attackers using data miners. High-utility itemset mining (HUIM) is an extension to frequent itemset mining (FIM) which deals with business data in the form of transaction databases, data that is also in danger of being stolen. To deal with this, a number of privacy-preserving data mining (PPDM) techniques have been introduced. An important topic in PPDM in the recent years is privacy-preserving utility mining (PPUM). The goal of PPUM is to protect the sensitive information, such as sensitive high-utility itemsets, in transaction databases, and make them undiscoverable for data mining techniques. However, available PPUM methods do not consider the generalization of items in databases (categories, classes, groups, etc.). These algorithms only consider the items at a specialized level, leaving the item combinations at a higher level vulnerable to attacks. The insights gained from higher abstraction levels are somewhat more valuable than those from lower levels since they contain the outlines of the data. To address this issue, this work suggests two PPUM algorithms, namely **MLHProtector** and **FMLHProtector**, to operate at all abstraction levels in a transaction database to protect them from data mining algorithms. Empirical experiments showed that both algorithms successfully protect the itemsets from being compromised by attackers.

## 1 Introduction

Frequent Itemset Mining is a technique that focuses on finding and discovering frequently occurring combinations of items (of arbitrary size) that appear in a transaction database [1]. Frequent itemsets have been applied in various research areas, including market basket analysis [2], recommendation systems [3, 4], and bioinformatics [5, 6], IoT [7].

For instance, in the context of market basket analysis, each row in the transaction database table represents a unique shopping basket. Transactions contain subsets of purchased items. Frequent itemsets represent sets of items that customers frequently purchase together [2]. For example, the itemset Fries,Ketchup indicates that customers who buy Fries often also purchase Ketchup. Since generating all possible combinations of items in a large transaction database is impractical, FIM aims to find only those item combinations that have a frequency (support) that meets a certain threshold, specified by the user. However, traditional frequent itemset mining approaches rely on the following three conditions [2], which may not be realistic: (a) the binary occurrence assumption; (b) the equal item importance assumption; and (c) ignoring item relationships.

The concept of High-Utility Itemset Mining (HUIM) was proposed [8] to overcome limitations (a) and (b) in the traditional FIM task. HUIM aims to discover item combinations that generate high overall profit or utility in the transaction database. HUIM is an extension of FIM that considers the utility of items instead of their frequency. This is particularly useful in scenarios where the quantity and profitability of items are important considerations.

In HUIM, two types of utilities are typically employed [8]. The first one is internal utility. It represents the quantity of an item purchased within a transaction. For example, in a grocery transaction the internal utility of Bread could be two loaves. The second is external utility. It represents the profitability or value of an item. For instance, the external utility of Bread could be $2 per loaf.

High-Utility Itemsets (HUIs) represent sets of items that often appear together in transactions and generate a total profit that exceeds a user-specified threshold. These HUIs provide valuable insights into customer purchasing patterns and can be utilized in various business strategies, such as: cross-selling, promotional planning, promotional planning, and so on.

In today’s interconnected world, organizations across various sectors, from commercial enterprises to government agencies, are increasingly engaging in data sharing practices. This trend is driven by the recognition that sharing data can unlock valuable insights, enabling organizations to identify global trends, optimize operations, and enhance their overall effectiveness. This new era of collaboration and data sharing has been facilitated by advances in information technology, which have made it easier and more efficient for organizations to exchange and analyze data. However, this increased data sharing also introduces new security challenges, as shared data becomes accessible to a wider range of entities.

Data mining techniques can be employed by partners or competitors to analyze shared data, potentially uncovering sensitive or strategic information that could be used to gain a competitive advantage, disrupt business operations, or even compromise information security. Some of the potential threats posed are unethical data mining, competitive intelligence, and fraudulent activities.

To address these critical issues, the concept of Privacy-Preserving Data Mining (PPDM) was proposed [9, 10]. PPDM techniques aim to protect sensitive data while still enabling the extraction of useful knowledge from it. In traditional PPDM approaches, the frequency of sensitive patterns is reduced below a user-defined threshold to achieve privacy protection. This is often achieved by removing sensitive transactions or related items from the database.

However, for sensitive data related to high-utility itemsets (which are considered valuable and need to be protected before the database is published or shared), it is necessary to reduce their utility values rather than their frequency. This is because reducing their frequency could lead to the loss of valuable insights. Privacy-Preserving Utility Mining (PPUM) emerged as an extension of PPDM algorithms to address this challenge [11, 12]. PPUM focuses on protecting sensitive information in databases by combining PPDM techniques with utility-based data mining methods.

To address limitation (c) from FIM, researchers have proposed methods that analyze item correlations at multiple abstraction levels. By considering the hierarchical nature of data, these methods can uncover more meaningful and insightful patterns. Consider the itemset Fries,Ketchup. Traditional FIM might identify this as a frequent pattern. However, multi-level abstraction analysis could reveal a more general pattern: Food,Condiment. This higher-level pattern captures the broader relationship between food items and their accompanying condiments. Considering multiple abstraction levels offers several benefits, such as uncovering patterns that are not apparent at a single abstraction level, providing a more comprehensive understanding of the data, and generating domain-specific insights by considering the hierarchical structure of data relevant to the specific domain.

Since 2017 HUIM has also been leveraged to work with databases containing multiple abstraction levels [13]. For the sake of simplicity, this type of database is referred to as a hierarchical database, while this new mining task can be referred to as Generalized HUIM (GHUIM).

Generalized HUI discovery has emerged as a promising approach for PPUM in the context of hierarchical data. Traditional PPUM algorithms often hide sensitive itemsets from transactions by adjusting their respective quantities or completely removing them from the transactions in regular databases. Although these approaches have advantages in terms of mining time and memory footprints, they cannot work with hierarchical databases. Furthermore, when applied to hierarchical databases, traditional PPUM approaches ignore items from higher abstraction levels. These sensitive items are still vulnerable and exploitable to data miners as they aggregate items from lower levels. For example, by allowing the itemsets *{Fries,Ketchup}* or *{Food,Condiment}* to be extracted from data miners might provide sensitive insights to the exploiters.

By incorporating hierarchical data mining techniques, PPUM algorithms can effectively protect sensitive data while still allowing the extraction of valuable utility patterns that span multiple abstraction levels. This opens up new possibilities for data analysis and knowledge discovery in various domains where hierarchical data structures are prevalent, while still keeping the sensitive data from undesired exploits.

To our knowledge, no approaches in PPUM have been proposed to work with hierarchical databases. Existing PPUM methods typically focus in traditional transaction database, without considering the generalization information among items. This information is important and provides useful knowledge for real-world applications. Thus, existing PPUM approaches cannot capture the inherent relationships and dependencies present in hierarchical data. This leaves a critical gap in addressing privacy concerns when sharing or publishing hierarchical data, which is prevalent in various domains. This distinction highlights the novelty of our work: we are the first to address the specific challenges of privacy-preserving utility mining in the context of hierarchical databases, moving beyond the limitations of existing approaches.

This work focuses on developing techniques for hiding user-specified sensitive multi-level high-utility itemsets (SML-HUI) in hierarchical databases. The proposed approach involves adjusting the utility values of SML-HUIs below a user-defined minimum utility threshold (denoted *ξ*) to protect sensitive information while preserving the overall utility of the data. The main contributions of this work are as follows.

- Developing strategies to select target sensitive items while considering the hierarchical structure of the database.
- Adopting the strategies to hide SML-HUIs from hierarchical databases. resulting in two novel algorithms, **MLHProtector** and **FMLHProtector**.
- The algorithms are then evaluated based on the several PPDM and PPUM criteria to demonstrate their effectiveness in hiding SML-HUIs while trying to preserve the original database structure.

The first algorithm, **MLHProtector**, hides the SML-HUIs by lowering their utility and prunes them off the transactions to completely hide them from extractors. The second algorithm, **FMLHProtector** employs faster utility reduction strategy to hide the SML-HUIs without removing them from the database, thus preserving the integrity of the database.

The remainder of this paper is structured as follows. The next section is a review of the literature related to PPUM. Section 3 presents the foundations of both HUIM and PPUM. Section 4 proposes both the core strategies and approaches used to efficiently carry out the multi-level high-utility itemset mining while preserving the privacy of the hierarchical databases. Section 5 presents the results of the experiments that were used to evaluate the proposed approaches from various perspectives. Finally, conclusions and future research directions are discussed in Section 6.

## 2 Related work

This section discusses recent works related to the scope of this work. They are grouped into three categories, ranging from FIM to HUIM and PPUM.

### Generalized FIM

Since FIM was first proposed, several approaches have been introduced, and the concept of item categorization was also suggested. The structure that stores the categorization of items is called a **taxonomy**. The Cumulate algorithm [14] which proposed by Skirant and Agrawal to address this new mining task. Hipp et al. introduced an algorithm called Prutax [15], combines item generalization with a vertical database format. It also employs two new strategies to prune unpromising candidates and extract frequent itemsets across abstraction levels in the database.

Sriphaew and Theeramunkong proposed the SET algorithm [16], adopting the set-enumeration mechanism to explore the search space. It combines constraints on generalized itemsets to speed up the mining phase. Pramudiono et al. suggested the FP-tax algorithm [17] based on the pattern-growth approach in 2004. It traverses the pattern-growth tree in both directions to generate generalized association rules. In 2009, Vo and Le proposed an efficient approach called MMS_Git-tree [18] to discover generalized association rules using only a single database scan. A framework known as CoGAR [19] was introduced by Baralis et al. and this utilizes several taxonomy structures combined with a number of constraints to generate generalized association rules.

Recently, two approaches dealing with frequent weighted utility itemset mining in hierarchical database were suggested by Nguyen et al. in 2022 [20]. The algorithms are MINE_FWUIS and FAST_MINE_FWUIS, and they adopt an extended version of the dynamic bit vector structure to efficiently address the mining task.

### High-utility itemset mining

As stated in the previous section, HUIM is an extension of FIM that aims to address its drawbacks. Since it was first proposed in 2004 by Yao and Hamilton [8], HUIM has attracted several studies to improve its mining performance via many efficient strategies and techniques. Some notable approaches in HUIM include the Two-Phase [21], HUI-Miner [22], FHM [23], EFIM [24], HMiner [25], and iMEFIM methods [26].

The first complete algorithm to perform the HUIM task was Two-Phase [21], which was proposed by Liu and Qu in 2005. The authors also introduced an upper bound called TWU (Transaction Weighted Utilization) to prune unpromising candidates from the search space, saving mining time and memory space. Later algorithms all rely on this upper bound to speed up the mining task.

However, Two-Phase, as its name implies, completes the extractions of HUIs in two stages [8] and thus consumes a large amount of time and memory. To address the drawbacks of algorithms based on the Two-Phase model, Liu et al. proposed the HUI-Miner algorithm [22]. This is the first single-phase HUIM algorithm. In addition, the authors introduced several novel and efficient techniques such as an upper bound known as the remaining utility, using the utility-list structure. These techniques are heavily utilized in later approaches. However, the complexity cost to join two utility-lists is expensive. As such, Fournier-Viger et al. proposed the FHM algorithm in 2014 [23]. The algorithm comes with a pruning strategy called EUCP. It utilizes a structure known as EUCS, which removes all extensions of an unpromising candidate, saving a significant amount of time and memory [23].

In 2017, Zida et al. proposed an algorithm named EFIM to handle the HUIM task [24]. The authors introduced a series of high-performance strategies and techniques, such as local utility, sub-tree utility, HDP, HTM, and so on. In the same year, Krishnamoorthy proposed the HMiner algorithm [25]. The author proposed a modified version of the utility-list called Compact Utility-List (CUL) to include both closed and non-closed utility. The algorithm also adopts previous efficient pruning strategies such as TWU, LA-Prune, EUCP, and others to improve the mining performance [25].

An approach called iMEFIM [26] was introduced by Nguyen et al. in 2019. This is an extension of the EFIM algorithm to address its drawback when working on dense databases. The P-Set structure was developed to address the high database scan cost of EFIM. In addition, this is also the first algorithm in HUIM to address the dynamic property of the utility measure [26].

Two approaches based on pattern-growth model were recently announced by Wang and Wang in 2021 [27]. The algorithms are HUIL-TN and HUI-TN, and they utilize a tree structure called TN-tree to avoid the candidate generation phase.

In 2023 Qu et al. proposed the HAMM algorithm [28]. This single-phase algorithm combined the pattern-growth tree with the prefix tree, utility vector and several optimizations to speed up the mining process.

Several works to work on dynamic database environment were also introduced, such as dynamic profits, negative utilities, or incremental databases. Some notable works are [29–33]. In addition, there are variations of the high-utility itemset mining task, such as utility occupancy utility itemsets [34–36], high average utility itemsets [37–39], etc.

### Privacy-preserving itemset mining

The main goal of data mining is to reveal hidden knowledge within data. However, during the process of revealing such information sensitive data can also be extracted unexpectedly. As such, several approaches have been introduced to address the privacy-related problems.

The first study to systematically survey this problem was from Fayyaad et al. in 1996 [40]. Later, Lindell et al. suggested an ID3-based approach to solve the problem of multi-party computation [41]. On 2004 Agrawal et al. introduced a method to transform a transaction database into an anonymized version of the original, condensing and grouping data before applying any data mining methods [42]. A border-based approach was introduced in 2005 by Sun et al. to determine the border value for sensitive frequent itemsets [43]. The approach then decides a proper value that is then lowered to hide sensitive itemsets. Li et al. proposed an approach based on the *k*D-tree structure to recursively partition the database into smaller database [44]. Sensitive itemsets are then hidden based on the average values of the smaller partitions.

In PPDM, algorithms to hide sensitive itemsets from the mining process must consider two primary factors: the hiding effect and side effects. Balancing between the two factors is an important task. Generally, data loss will occur when performing database sanitization, and this factor must be evaluated. Bertino et al. suggested an approach to measure three primary factors of side effects when performing the privacy-preserving data mining task [10]. They are **hiding failure** (HF), **missing cost** (MC) and **artificial cost** (AC). If the database sanitization process failed to hide sensitive data, this would allow the attackers to exploit them. The HF factor is used to evaluate the performance of this process. The sanitization process may also cause the loss of some non-sensitive but frequent itemsets. This is evaluated using the MC factor. In contrast, some itemsets which were previously infrequent in the original database might become frequent in the sanitized database. This would yield low accuracy of the mining process and it is evaluated using the AC factor.

PPUM is considered as an extension to PPDM, which considers the utility of items [11]. Similar factors in PPDM are also adopted in PPUM to measure the privacy-preserving performance of proposed approaches [12], and these are **database structure similarity** (DSS), **database utility similarity** (DUS) and **itemset utility similarity** (IUS).

However, studies focusing on PPUM are still limited, although some notable works include those on HHUIF and MSICF [11]; HMAU [45]; FPUTT [46]; MSU-MAU and MSU-MIU [12]; SMAU, SMIU and SMSE [47]; MinMax and Weighted [48]; SMRF, SLRF and SDIF [49]; and FULD [50].

Yeh and Hsu are the pioneers in the field of PPUM [11]. The authors suggested two approaches, HHUIF and MSICF, to solve this new PPUM task in 2010 [11]. Both algorithms hide sensitive HUIs by lowering their utilities. However, they use different techniques to select target items. HHUIF selects the item whose utility is the largest among transactions, while MSICF selects the items that have the highest occurrence frequency among sensitive HUIs to reduce their quantities.

An algorithm named HMAU was proposed by Lin et al. in 2014, and this hides sensitive high-utility itemsets via transaction deletion. Yun and Kim proposed a tree-based approach called FPUTT that performs fast database perturbation to prevent the exploitation of sensitive information [46]. In 2016, Lin et al. proposed two PPUM algorithms, namely MSU-MAU and MSU-MIU [12] which respectively select items having the highest and lowest utility from transactions containing sensitive HUIs to perform adjustments. The mentioned PPUM factors are also proposed in this work [12]. In 2020 Liu et al. proposed a series of three algorithms named SMAU, SMIU and SMSE to protect sensitive data [47]. The major differences among the algorithms are the sensitive item selection strategies: selecting items with maximum utility first, minimum utility first and minimum side effects first, respectively. In 2022, two algorithms called MinMax and Weighted were presented by Jangra and Toshniwal to mask sensitive information from HUI miners [48]. A series of three algorithms were also put forward by Ashraf et al. in 2023 [49]. Similar to previous algorithms, the authors introduced three different strategies for sensitive item selection, SMRF (favoring the most real item sensitive utility), SLRF (favoring the least real item sensitive utility) and SDIF (favoring the most desirable Item). Yin and Li presented the FULD algorithm in 2023 (Yin & Li, 2023), and this is based on a utility-list dictionary allowing fast sensitive lookup, combines with side-effect reduction strategies.

PPDM and PPUM are still important due to increasing concerns about data privacy. A recent work [51] proposed an algorithm called MSU-MSI to hide sensitive itemset from sensory data obtained from IoT devices. Also in 2024, Gui et al. proposed two methods to secure rare itemsets during mining [52]. The algorithms are LT-MIN and LT-MAX, aiming to reduce the side effects of the database sanitization process. Le et al. introduced an algorithm called H-FHAUI [53] to hide frequent high average utility itemset, combining both PPDM and PPUM.

However, to the best of our knowledge, none of the previous works in the field PPUM consider the generalization of items in the transaction databases. Thus, this type of database is still vulnerable from attackers seeking to gain sensitive information. The aim of the current work is thus to propose solutions to close this gap in PPUM.

## 3 Preliminaries

This section presents basic and core definitions of the HUIM task and provides the problem statement, which is the main goal of this work.

**Definition 1** ***Transaction database*** [22].

*Let* $I=\{i_{1},i_{2},\ldots ,i_{n}\}$ *be a universal set of all distinct items, a transaction database in HUIM is a multiset of transactions, denoted as* D , $D=\{T_{1},T_{2},...,T_{m}\}$. *Whereas T*_*k*_ $\left(T_{k}\subseteq I,1\leq k\leq m\right)$ *is a transaction. Each T*_*k*_ *has a unique transaction identifier (TID) k and consists of the following information*.

- *A set of items* $\{j_{1},j_{2},\ldots ,j_{v}\}\subseteq I$ , (1 ≤ *v* ≤ *n*).
- *A set of values called internal utility of each respective items j*_*v*_ *in T*_*k*_, *and is denoted as iu*(*j*_*v*_,*T*_*k*_).

In addition, each item in I is also associated with a positive integer, called external utility. This value is denoted as *eu*(*i*_*k*_), (1 ≤ *k* ≤ *n*).

For example, Table 1 illustrates a sample transaction database, which will be used throughout this work as a running example. Considering *T*_6_, this transaction has a unique identifier 6; it contains three items A,C and E; the respective internal utilities of these items are 3,4 and 4. In addition, Table 2 presents the list of all external utilities for the set I used in this transaction database. Considering item *B*, its external utility is *eu*(*B*) = 1.

**Table 1:** A sample transaction database.

TID	Items	Internal Utilities
T_1_	A, C	3, 7
T_2_	A, C, D, E	7, 2, 3, 5
T_3_	A, B, C, D, E	8, 3, 6, 8, 9
T_4_	B, C, D, E	4, 5, 3, 7
T_5_	A, D	9, 9
T_6_	A, C, E	3, 4, 4
T_7_	B, C, E	7, 7, 8
T_8_	C, D	2, 4
T_9_	C, D, E	8, 3, 7
T_10_	A, C, D	4, 7, 9

**Table 2:** External utilities of items from Table 1.

Items	A	B	C	D	E
**External Utility**	2	1	2	1	2

**Definition 2** ***Utility computations*** [21].

- *The utility of an item* i∈I *in transaction T*_*k*_, *denoted as u*(*i*, *T*_*k*_), *is determined as u*(*i*, *T*_*k*_) = *iu*(*i*, *T*_*k*_) × *eu*(*i*).
- *The utility of an item* i∈I *in the whole database* D , *denoted as u*(*i*), *is computed as* $u\left(i\right)=\sum_{i\in T_{k}\wedge T_{k}\in D}u\left(i,T_{k}\right)$.
- *The utility of an itemset* P , P⊆I, *in transaction T*_*k*_, *denoted as* $u(P,T_{k})$, *is calculated as* $u\left(P,T_{k}\right)=\sum_{i\in P\wedge P\subseteq T_{k}}u\left(i,T_{k}\right)$.
- *The utility of an itemset* P , P⊆I, *in the whole database* I, *denoted as* u(P), *is determined as* $u\left(P\right)=\sum_{P\subseteq T_{k}\wedge k\in t\left(P\right)}u\left(P,T_{k}\right)$.
- *The utility of a transaction T*_*k*_,$T_{k}\in D$, *denoted as TU*(*T*_*k*_), *is determined as* $TU\left(T_{k}\right)=\sum_{i\in T_{k}\wedge T_{k}\in D}u\left(i,T_{k}\right)$.

Whereas t(P) denotes the set of all TIDs containing the itemset P. For example, using database *D* in Tables 1 and 2, then:

- *u*(*A*, *T*_3_) = 2 × 8 = 16.
- *u*({*A*, *C*}, *T*_3_) = *u*(*A*, *T*_3_) + *u*(*C*, *T*_3_) = 16 + 12 = 28.
- *u*({*A*, *C*}) = *u*({*A*, *C*}, *T*_1_) + *u*({*A*, *C*}, *T*_2_) + *u*({*A*, *C*}, *T*_3_) + *u*({*A*, *C*}, *T*_6_) + *u*({*A*, *C*}, *T*_10_) = 20 + 18 + 28 + 14 + 22 = 102.
- *TU*(*T*_5_) = *u*(*A*, *T*_5_) + *u*(*D*, *T*_5_) = 2 × 9 + 1 × 9 = 27.

It could be worth noting that the utility measure **does not** satisfy the downward closure property (also known as the Apriori property). Hence, HUIM approaches cannot directly use this measure or any efficient pruning strategies, which were utilized in FIM, to optimize the problem’s search space.

To reduce the search space of this mining task, an upper bound was proposed by Liu et al. in 2005 [21]. The upper bound is called Transaction Weighted Utilization (TWU) and is defined as follows.

**Definition 3** ***Transaction Weighted Utilization*** [21].

*Transaction Weighted Utilization of an itemset* P , P⊆I, denoted as TWU(P), is determined as: $TWU\left(P\right)=\sum_{P\subseteq T_{k}\wedge k\in t\left(P\right)}TU\left(T_{k}\right)$.

This upper bound is proved that it satisfies the Downward Closure Property (DCP) [12], and thus can be used to safely prune all unpromising candidates from the search space, saving both mining time and memory usage for the mining task.

For example,

- *TWU*({*A*}) = *TU*(*T*_1_) + *TU*(*T*_2_) + *TU*(*T*_3_) + *TU*(*T*_5_) + *TU*(*T*_6_) + *TU*(*T*_10_) = 20 + 31 + 57 + 27 + 22 + 31 = 188.
- *TWU*({*A*, *D*}) = *TU*(*T*_2_) + *TU*(*T*_3_) + *TU*(*T*_5_) + *TU*(*T*_10_) = 31 + 57 + 27 + 31 = 146.

**Definition 4 *High-Utility Itemset*** [21].

*Given a transaction database* D *and an itemset* P(P⊆I) . P is a high utility itemset (HUI) if and only if u(P) is no less than a user-specified minimum utility threshold: u(P)≥ξ.

**Definition 5 *Database’s taxonomy structure*** [13].

*Let* S *be a tree defined on* I *of transaction database* D . S *is called a taxonomy of items in* D *and has the following properties*:

- *All the leaf nodes in* S *represent the items in* I *(specialized nodes/items)*.
- *The inner nodes in* S *aggregate the specialized node into a generalized node g at a higher abstraction level in this taxonomy. The set of all generalized nodes in* S *denoted as* G .
- *Each specialized item* i∈I *can be generalized into one and only one direct generalized item* g∈G , *i.e. one level above i*. *The same term is applied to all generalized item g*, *recursively*.
- *The set of all descendant nodes of g*, *denoted as DESC*(*g*), *contains all the specialized/leaf nodes of g and* DESC(g,S)⊆I.
- *Let* G *be a multi-level itemset in* D *using* S , *then the set of all descendant nodes of* G *in* S *is determined as* DESC(G,S)={v∣v∈|v∈I∧v∈DESC(g,S)∧g∈G}.

To efficiently represent the taxonomy structure, in this work, we store the taxonomy as pairs of 〈*key*, *value*〉 to map child items to their direct respective parent. This can be achieved by using a hash map or a dictionary. Thus, speeding up the lookup and traversal on the taxonomy structure.

Definition of a multi-level itemset is provided in **Definition 7** below.

**Fig 1:**
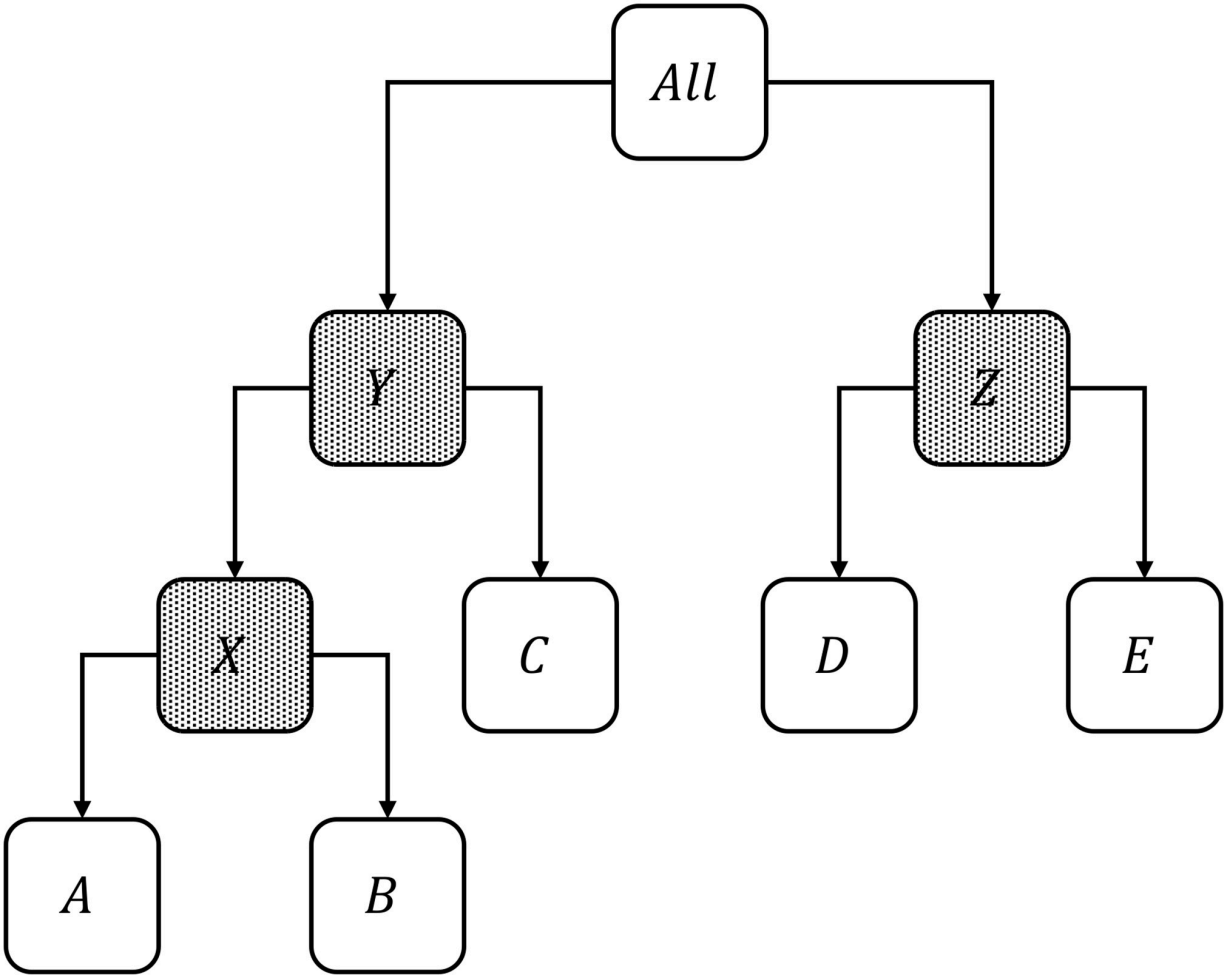
Sample taxonomy of the transaction database in Table 1.

For example, Fig 1 depicts a sample taxonomy structure S of the transaction database D given in Table 1. In this taxonomy, the specialized items *D* and *E* are generalized into an item named *Z*, or the general item *Z* aggregates two specialized items *D* and *E*. The root node (denoted as *All*) aggregates all the generalized items in D.

**Definition 6 *Level of an item*** [13].

*Let* D *be a transaction database, with the taxonomy* S *defined on* D *and an item* g∈S . *The level of g in* S *is defined as the number of edges needed to reach g from the root node of* S, *denoted as* level(g,S).

*For example, using the taxonomy in* Fig 1, *the level of the general item Y is* level(Y,S)=1 *and* level(A,S)=level(B,S)=3.

**Definition 7 *Multi-level Itemset*** [13].

*Given an itemset* G , G *is a multi-level itemset if and only if* {∀u,v∣u,v∈G∧level(u)=level(v)}.

*A multi-level itemset is an itemset containing items from the same abstraction level. It could be worth noting that **Definition 7** also applies to the specialized items, whereas their levels equal to zero. In the case of the taxonomy is empty, the database is reverted back to traditional transaction database*.

*For example, using the taxonomy in* Fig 1 *the itemset* {*Y*, *Z*} *is considered a multi-level itemset, since* level(Y,S)=level(Z,S)=1. *However, the itemset* {*X*, *Z*} *is not a multi-level itemset since* level(X,S)≠level(Z,S).

**Definition 8 *Taxonomy-based utility computations*** [13].

- *The utility of a general item g in transaction T*_*k*_ *in database* D , *using taxonomy* S *is defined as* $u\left(g,T_{k}\right)=\sum_{i\in DESC\left(g,S\right)\wedge i\in T_{k}\wedge T_{k}\in D}iu\left(i,T_{k}\right)\times eu\left(i\right)$.
- *The utility of a general itemset* G *in transaction T*_*k*_ *in database* D , *using taxonomy* S *is defined as* $u\left(G,T_{k}\right)=\sum_{g\in G}u\left(g,T_{k}\right).$
- *The utility of a general itemset* G in D, *using taxonomy* S *is defined as* $u\left(G\right)=\sum_{g\in G\wedge k\in t(G)}u\left(g,T_{k}\right).$

*Whereas* $t\left(G\right)=\{k|\forall g\in G:\exists v\in T_{k}\wedge v\in DESC\left(g,S\right)\wedge T_{k}\in D\}$

For example, considering the transaction database D in Table 1, using the taxonomy S in Fig 1, then:

- The utility of general item *Z* in *T*_3_ based on S is: *u*(*Z*, *T*_3_) = *u*(*D*, *T*_3_) + *u*(*E*, *T*_3_) = 1 × 8+ 2 × 9 = 26.
- The utility of itemset {*X*, *E*} in *T*_3_ based on S is: *u*({*X*, *E*}, *T*_3_) = *u*(*X*, *T*_3_) + *u*(*E*, *T*_3_) = *u*(*A*, *T*_3_) + *u*(*B*, *T*_3_) + *u*(*E*, *T*_3_) = 37.
- In addition, the utility of {*X*, *E*} in the whole database D based on S is *u*({*X*, *E*}) = *u*({*X*, *E*}, *T*_2_) + *u*({*X*, *E*}, *T*_3_) + *u*({*X*, *E*}, *T*_4_) + *u*({*X*, *E*}, *T*_6_) + *u*({*X*, *E*}, *T*_7_) = 24 + 37 + 18 + 14 + 23 = 116.

**Definition 9 *Multi-level HUI*** [13].

*Given a transaction database* D , *a minimum utility threshold ξ and taxonomy* S. *An itemset* G *is a multi-level HUI (MLHUI) if and only if it is a multi-level itemset and its utility is no less than the ξ threshold:* u(G)≥ξ.

**Definition 10 *The Multi-level High-Utility Itemset Mining*** [13].

*Given a transaction database* D *and a user-specified minimum utility threshold ξ*. *The task of High-Utility Itemset Mining (HUIM) is to extract all the itemsets whose utility satisfies the ξ threshold:* HUIs←{P∣u(P)≥ξ}.

*The HUIM task is also extended to work with hierarchical transaction database* D *using taxonomy* S . *The complete set of discovered multi-level HUIs from* D *using* S *is denoted as MLHUIs* ←{P∣u(P)≥ξ}.

**Definition 11 *Sensitive High-utility Itemset*** [11].

*A MLHUI that exposes sensitive information in a transaction database* D *after* D *is published is called a sensitive MLHUI (SML-HUI). The set of all SML-HUIs is specified by the user*.

**Definition 12 *Side-effect factors*** [10].

*Given* $S=\{s_{1},s_{2},\ldots ,s_{k}\}$ *as the set of sensitive MLHUIs that need to be hidden from database* D , MLHUIs* *denotes the number MLHUIs in* D *post-sanitized, then:*

- *Let HF denote the number of sensitive HUIs the sanitization process failed to hide and are still present in the database*. *HF is determined as:* $HF=\alpha =S\cap MLHUIs^{*}$.
- *Let MC denote the number of non-sensitive MLHUIs that would be hidden from* D *post-sanitizing. MC is determined as* $MC=\tilde{S}\backslash MLHUIs^{*}$ . *Whereas* $\tilde{S}$ *is the set of non-sensitive MLHUIs*, $\tilde{S}=\beta =MLHUIs\backslash S$.
- *Let AC denote the number of itemsets that are non-HUIs but become HUIs in* D *post-sanitizing. AC is determined as AC* = *γ* = MLHUIs*∖*MLHUIs*.
  *Besides the above-mentioned factors in PPDM, Lin et al. also introduced the measures to evaluate the similarity between the original and the sanitized databases. The measures are DSS (Database Structure Similarity), DUS (Database Utility Similarity) and IUS (Itemset Utility Similarity). Details of the measures are provided in the related work* [12].

**Definition 13 *The PPUM task*** [11].

*Let HS be a set of sensitive MLHUIs. The goal of PPUM is to hide as many items as possible in the set HS while trying to minimize the cost of the side effect factors (HF*, *MC*, *AC in PPDM*; *DSS*, *DUS and IUS in PPUM)*.

## 4 Proposed approaches

### Problem statement

Given a transaction database D and its associated taxonomy S, a set MLHUIs contains all the multi-level high-utility itemsets obtained from a MLHUIM algorithm at a specified *ξ* threshold. A set S⊆MLHUIs, $S=\{s_{1},s_{2},...,s_{k}$} containing all the sensitive MLHUIs that need to be hidden and is specified by the user.

The goal of PPUM is to construct a database D’ based on the aforementioned input parameters to hide the set S from MLHUIM algorithms.

### Hiding sensitive multi-level high-utility itemsets from a hierarchical database

The scope and goals of this work are to propose approaches to solve the PPUM task on hierarchical databases. The algorithms, namely **MLHProtector** and **FMLHProtector**, are based on the HHUIF algorithm [11]. The techniques used in HHUIF are extended and leveraged to work with taxonomy-based transaction databases.

The three properties of HHUIF addressed in this work are:

- HHUIF lowers the utility value of target sensitive itemset below the *ξ* threshold, in order to hide them from HUIM algorithms.
- HHUIF also completely removes the target sensitive itemset from the transactions. This leads to a significant difference between the original database and the sanitized database.
- In addition, HHUIF also consumes a large amount of time to search and hide the target sensitive itemset.

**MLHProtector** and **FMLHProtector** are based on the basic ideas of HHUIF. The algorithms perform the database sanitization process by adjusting the utility of SML-HUIs so that it is lower than the *ξ* threshold. The internal and external utility of both specialized and generalized items in SML-HUIs are considered. In general, the core steps of both algorithms are as follows.

- Applying a MLHUIM algorithm on the hierarchical database D to obtain the complete set of MLHUIs at the specified *ξ* threshold.
- From the discovered MLHUIs, the user provides a set of sensitive MLHUIs that need to be hidden.
- Applying a PPUM on the original hierarchical database to hide all the SML-HUIs with minimal impacts on the original database.
- Both proposed algorithm, **MLHProtector** and **FMLHProtector** can operate on hierarchical transaction databases to guard them from exploiting sensitive high utility itemsets.
- The **MLHProtector** algorithm lowering the quantity of the items in all SML-HUIs and eventually, might remove the items that reach zero quantity. However, this would affect the intergity of the original databases.
- The **FMLHProtector** algorithm also lowering the quantity of all sensitive leaf nodes in all SML-HUIs. However, the retain the original structure of the sanitized databases, the algorithm prevent the removal of items from transactions. In addition, the set of all leafs nodes is sorted before performing the sanitizing process to boost the performance.

To achieve the goals, the algorithms need to process the original hierarchical database D using taxonomy S starting from the specialized items at the leaf nodes of S. The result obtained is the sanitized database D’, preventing sensitive knowledge from being exploits while retaining insensitive data. The sanitizing process is independent of the taxonomy S from the original database D.

To perform the expansions from a generalized item back to the list of all its descendants and their respective utility values, the following property is employed.

**Property 1. Descendants’ expansion of a generalized item.**

Let g∈G be a generalized item, a transaction database G, the set of all specialized items I, and the respective taxonomy S of D. Descendants’ expansion of *g* can be obtained using the set DESC(g,S).

**Proof:**

- Given a specialized item i∈I, i is called a descendant of *g* using taxonomy S. Thus i∈DESC(g,S) based on **Definition 5**.(*)
- In addition, **Definition 5** ensures each descendant node i∈I belongs to one and only one generalized item g∈G (**)

Based on (*), for any specialized item v∈I, if v∈DESC(g,S) then there exists a reverse mapping between the set DESC(g,S) back to *v*. In addition, based on (**) this mapping is unique. Thus, **Property 1** holds.

**Property 2. Descendant’s utility expansion of a generalized item.**

Let D be a transaction database accompanied with taxonomy S, g∈G be a generalized item, DESC(g,S) be the set of all descendants of *g*, then the following holds.

- The utility of all descendants of *g* in transaction *T*_*k*_ can be computed using **Definition 10**.
- The utility of all descendants of *g* in D using taxonomy S can be directly determined using **Definition 10**.

**Property 1** and **Property 2** are adopted by both **MLHProtector** and **FMLHProtector** to trace back all descendants of any generalized item, using the taxonomy S (**Definition 5**). Furthermore, the utility of all descendants can also be obtained using **Definition 10**.

Specifically, the properties are employed in the **MLHProtector** algorithm at line #3 (**Algorithm 1**). It is also adopted in the **FMLHProtector** algorithm at line #2 (**Algorithm 2**). The purpose of this property is to transform all generalized items back to its specialized form, containing only specialized items.

**Fig 2:**
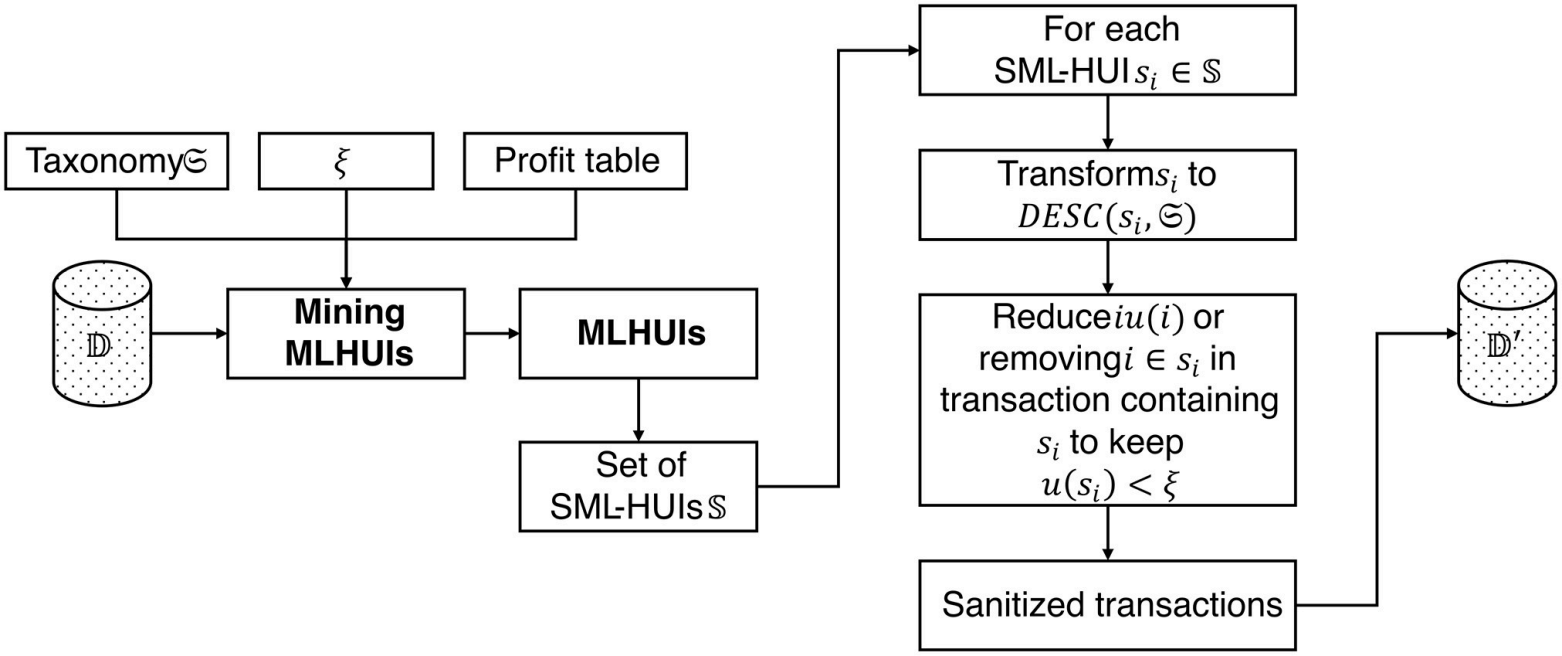
The architecture of both MLHProtector and FMLHProtector.

Based on this, each SML-HUI must be converted back to the set of specialized items based on **Definition 9**. Thus, both **MLHProtector** and **FMLHProtector** focus on reducing the utility value of the specialized items using several techniques to keep them lower than the threshold *ξ*. Specifically, **MLHProtector** combines both utility reduction and/or removal of sensitive MLHUIs, while **FMLHProtector** employs only utility reduction. Fig 2 depicts the shared architecture of both proposed algorithms, **MLHProtector** and **FMLHProtector**.

#### MLHProtector algorithm

The **MLHProtector** is based on the basic concepts of HHUIF and is designed to work with a hierarchical database enriched with the taxonomy S. Specifically, for each SML-HUI after being transformed back to its specialized form (containing sensitive leaf nodes), the algorithm performs the database sanitization process by adjusting the internal utility of the sensitive leaf nodes per transaction.

To achieve this, **MLHProtector** selects a sensitive leaf node with the highest utility value among transactions and adjusts its quantity. The process repeats for all remaining sensitive leaf nodes, until the utility value of the sensitive item become lower than the *ξ* threshold. The **MLHProtector** performs the hiding task for the rest of the set of SML-HUIs S until all the itemsets are completely hidden. The algorithm **MLHProtector** is presented in **Algorithm 1**.

**Algorithm 1:** MLHProtector algorithm

**Input:** D : transaction database; S: taxonomy; *ptable*: item’s profit table; S: set of sensitive MLHUIs that need to be hidden; *ξ*: minimum utility threshold.

**Ouput:** Sanitized transaction database in which the set SML-HUIs were completely hidden.

**1 for** *each* $S_{g}\in S$ **do**

**2**  *diff*_*g*_ = *u*(*S*_*g*_) − *ξ*

**3**  Transform *S*_*g*_ into $DESC(s_{g},S)$, denoted as *S*_*L*_.

**4**  **for** *each i*∈*S*_*L*_ **do**

**5**   *sum* ← ∑_*j*∈*t*(*S*_*g*_)_*u*(*i*, *T*_*j*_)

**6**   $diff_{i}=\frac{sum}{u(S_{i})}\times diff_{g}$

**7**   **while** *diff*_*i*_ > 0 **do**

**8**    *T*_*p*_ ← *argmax*_*p*∈*t*(*S*_*g*_)_*u*(*i*, *T*_*p*_)

**9**     $iu(i,T_{p})\leftarrow \{\begin{array}{l} 0,ifu(i,T_{p})\leq diff_{i}\\ iu(i,T_{p})-\lceil \frac{diff_{i}}{ptable_{i}}\rceil ,ifu(i,T_{p})>diff_{i} \end{array}$

**10**     $diff_{i}\leftarrow \{\begin{array}{l} diff_{i}-u(i,T_{p}),ifu(i,T_{p})\leq diff_{i}\\ 0,ifu(i,T_{p})>diff_{i} \end{array}$

   Update D based on *iu*(*i*, *T*_*p*_)

**11 return** D

A detailed explanation of the **MLHProtector** algorithm, as presented in **Algorithm 1**, is given below.

- For each SML-HUI *S*_*g*_ from the set S provided by the users (line #1), at line #2 the algorithm determines the difference *diff*_*g*_ between the utility of *S*_*g*_ and the specified *ξ* threshold.
- Line #3 transforms the itemset *S*_*g*_ into a form that contains only the leaf nodes, based on the taxonomy S and the set $DESC(S_{g},S)$. The transformed itemset of *S*_*g*_ is denoted as *S*_*L*_.
- A loop to scan through each sensitive leaf node i in *S*_*L*_ is carried out from lines #4 to #12.
- The utility of item *i* in all transactions that contain *S*_*L*_ is then computed at line #5.
- Line #6 computes the *diff*_*i*_ value, which is the reduced value of *i* to lower $S_{g}^{\prime }s$ utility below *ξ*.
- From lines #7 to #11, the algorithm reduces the utility of the sensitive leaf node *i* in all transactions containing *S*_*L*_ until *diff*_*i*_ ≤ 0.
  - At line #8, the algorithm selects a leaf node *i* in transaction *T*_*p*_ containing *S*_*L*_ such that *i* has the highest utility, denoted as (*i*, *T*_*p*_). This item *i* is the item that needs to be modified with regard to its internal utility in transaction *T*_*p*_ at line #9.
  - Line #9 adjusts the internal utility of *i* in *T*_*p*_ based on the following conditions:
    * If *u*(*i*, *T*_*p*_)<*diff*_*i*_, its internal utility is set to zero, and then the algorithm moves to the next item.
    * Otherwise, if *u*(*i*, *T*_*p*_)>*diff*_*i*_, its internal utility is reduced by an amount equal to $\left\lceil {\frac{diff_{i}}{ptable}}_{i}\right\rceil$. Whereas *ptable*_*i*_ denotes the external utility of *i*.
  - Similarly, line #10 re-evaluates the value of *diff*_*i*_ based on the utility of *i* in *T*_*p*_ against itself.
  - Finally, at line #11, the algorithm updates all the changes made on *i* into the original database D.
- The algorithm continues to process the next item in *S*_*L*_ until all the sensitive leaf nodes have their utility reduced. This would also, in turn, lower the overall utility of *S*_*L*_ below *ξ* threshold.
- The same operations are carried out until all the SML-HUIs in S are fully processed.
- Finally, the algorithm returns the sanitized transaction database D, denoted as D’.

#### FMLHProtector algorithm

**FMLHProtector** is an optimized version of **MLHProtector** to reduce the execution time of the PPUM task.

Based on the obseveration from the **MLHProtector** algorithm, the larger itemset, the more sensitive leaf nodes it contains. However, the algorithm process the itemsets in a simple mechanism: “First come, first served”. In the worst scenario, if smaller itemsets arrive first, then the algorithm needs longer execution time to process all the sensitive leaf nodes.

To address this problem, **FMLHProtector** use a strategy that sorts the set (or list) of all transformed sensitive MLHUIs $S_{SL}$ in the descending order of size of each itemset. Larger itemsets are the first to be processed, then to the smaller ones. This would help the algorithms select the itemset containing the most sensitive leaf nodes to be processed first. Once an itemset *S*_*L*_ is successfully hidden, all its related leaf nodes would have their utilities reduced. This would in turn increase the possibility of lowering the utility of other SML-HUIs. When a sensitive leaf node is successfully hidden, it will no longer need to be considered in the processing of next itemsets. Thus, speeding up the sanitization process and the whole algorithm.

**Algorithm 2:** FMLHProtector algorithm

**Input:** D : transaction database; S: taxonomy; *ptable*: item’s profit table; S: set of sensitive MLHUIs that need to be hidden; *ξ*: minimum utility threshold.

**Output:** D ’: Sanitized transaction database in which the set SML-HUIs were completely hidden.

**1 for** *each* $S_{g}\in S$ **do**

**2**  Transform *S*_*g*_ to $DESC(S_{g},S)$,denoted as *S*_*L*_.

**3**  Add *S*_*L*_ to the list $S_{SL}$ using the descending order of |*S*_*L*_|.

**4 for** *each* $S_{L}\in S_{SL}$ **do**

**5**  *diff*_*L*_ ← *u*(*S*_*L*_) − *ξ*; *diff*_*counter* ← *diff*_*L*_

**6**  **if** *diff*_*L*_ > 0 **then**

**7**   **for** *each i* ∈ *S*_*L*_ **do**

**8**    *sum* ← ∑_*p* ∈ *t*(*S*_*L*_)_*u*(*i*, *T*_*p*_)

**9**    $diff_{i}\leftarrow \frac{sum}{u(S_{i})}\times diff_{L}$

**10**    **for** *each p* ∈ *t*(*S*_*L*_) **do**

**11**     $rq\leftarrow \lceil \frac{diff_{i}}{ptable_{i}}\times \frac{u(i,T_{p})}{sum}\rceil$

**12**     $iu(i,T_{p})\leftarrow \{\begin{array}{l} 1,iu(i,T_{p}),-1\leq rq\\ iu(i,T_{p})-rq,ifu(i,T_{p})-1>rq \end{array}$

**13**     Update D based on *iu*(*i*, *T*_*p*_) *diff*_*counter*− = *rq* × *ptable*_*i*_

**14**     **if** *diff*_*counter* < 0 **then**

**15**      CONTINUE

**16 return** D as the sanitized database D’

A detailed explanation of the **FMLHProtector** algorithm as presented in **Algorithm 2** is given below.

- Lines #1 to #4 first perform the aforementioned strategy.
  - First, similar to the **MLHProtector** algorithm, each SML-HUI *S*_*g*_ in the set S will be transformed back to a representation that contains only the sensitive leaf nodes. Taxonomy S and the set of all descendant nodes of *S*_*g*_ will be utilized.
  - The transformed SML-HUI, which is denoted as *S*_*L*_, is then inserted into a data structure, with the ordering of descending order of the length of the itemsets.
- Lines #5 to #21 extract each SML-HUI *S*_*L*_ from the sorted list *S*_*SL*_ to process. Details are as follows.
  - Line #6 computes the delta between *ξ* and *u*(*S*_*L*_) and stores it as *diff*_*L*_. *diff*_*L*_ also acts as a processing bound for *S*_*L*_, denoted as *diff*_*counter*.
  - If *diff*_*L*_ is determined as a positive number, **FMLHProtector** then starts lowering the utility of each leaf nodes in *S*_*L*_ until it becomes a negative value. This occurs from lines #8 to #18. Using *diff*_*L*_ allows the algorithm to bypass the already processed sensitive leaf nodes, and thus speed up the algorithm.
  - The total utility of *i* in all transactions *T*_*p*_ that *i* occurred in is calculated and denoted as *sum*.
  - Line #9 determines the value *diff*_*i*_ for the item *i*.
  - Lines #11 to #15 perform the reduction of internal utility of item *i* following its transactions. The reduction amount is *rq*. To avoid the removal of *i* (internal utility equals to zero), its quantity at transaction *T*_*p*_ is set to 1 if *u*(*i*, *T*_*p*_) − 1 < *rq*, otherwise, its quantity is reduced by an amount of *rq* (Line #13).
  - The modified values of i are then reflected in D (Line #14).
  - Lines #15 and #16 update the value of *diff*_*counter* and test if the processing of *S*_*L*_ is done (*diff*_*counter* < 0). The algorithm then moves to process the next SML-HUIs in the list *S*_*SL*_.
- Finally, after the set S is completely hidden, **FMLHProtector** returns the sanitized database D, known as D’.

### Complexity analysis

Considering the **MLHProtector** algorithm, let *n* denotes the size of the set containing all SML-HUIs S, n=|S|; *m* denotes the maximum length of all itemsets contain in S and *k* denotes the total number of transactions containing all the itemsets in S. Then, the time complexity of **MLHProtector** in the worst case can be determined as *O*(*n* × *m* × *k*). The term *k* in practice can be very large, especially on dense and large databases. Thus, *k* significantly affect the runtime of the overall sanitizing process. In addition, the more SML-HUIs need to be hide, the higher time complexity of the algorithm.

For the **FMLHProtector**, the time complexity can be determined as follows. Let *n* be the number of sensitive leaf nodes contained in the set $S_{SL}$; *m* denotes the maximum length of all transformed itemsets contain in $S_{SL}$ and *k* be the total number of transactions containing all itemsets in $S_{SL}$. The sorting operation of this set has the average/worst time complexity of *O*(*nlog*_2_*n*), which only executed once. The worst time complexity of the FMLHProtector is approximately *O*(*nlog*_2_*n*) + *O*(*n* × *m* × *k*). Similar to the MLHProtector algorithm, FMLHProtector is also affected majorly from the *k* factor. However, since the set $S_{SL}$ was sorted in the descending order of each itemset’s length, longer itemsets would be processed first and thus, increase the performance of the sanitizing process as more items were already sanitized and thus can be skipped in later itemsets.

Overall, both proposed algorithms apply changes onto the original databases to sanitize them. They process the internal utility of the sensitive items per transactions. Thus, the number of changes made to the original database in the worst case is the total transactions that contain all SML-HUIs in the set S. Let *ω* be the number of affected transactions, *ω* can be determined as follows. $\omega =\sum_{S_{g}\in S}\left|t\left(S_{g}\right)\right|$, whereas *t*(*S*_*g*_) is the set of all transactions containing the itemset $S_{g}\in S$.

### Data availability and analysis

This work proposed two algorithms to carry out the PPUM task on hierarchical databases. To the best of our knowledge, the algorithm **MLHProtector** and **FMLHProtector** are the first algorithms proposed. During the development of the algorithms, several databases were obtained and analyzed, assisting the validation and verification of the algorithms. The databases used in this study are publicly available from the SPMF Open Source Data Mining Library (https://www.philippe-fournier-viger.com/spmf/index.php?link=datasets.php). The databases was used and analyzed in accordance with the terms and conditions outlined by the license provided at the SPMF library. Please note that any specific use or redistribution of the databases may require additional permissions or licenses from the original source. The databases are stored in plain text, comply with the SPMF format. The taxonomy structures are stored as list of pairs of (*child*, *parent*).

### An illustrative example

Considering the transaction database D given in Tables 1 and 2 and is transformed into the database presented in Table 3 and the taxonomy information provided in Fig 1. After running a multi-level high utility itemset miner at *ξ* = 88, the following ML-HUIs are obtained and provided in Table 4. Assuming the set of selected SML-HUIs is given in Table 5.

**Table 3:** Database D after computing the utility values of each item in transactions.

Internal utility					TID	Utility				
A	B	C	D	E		A	B	C	D	E
3		7			*T*_1_	6		14		
7		2	3	5	*T*_2_	14	4	3	10	
8	3	6	8	9	*T*_3_	16	3	12	8	18
	4	5	3	7	*T*_4_		4	10	3	14
9			9		*T*_5_	18			9	
3		4		4	*T*_6_	6		8		8
	7	7		8	*T*_7_		7	14		16
		2	4		*T*_8_			4	4	
		8	3	7	*T*_9_			16	3	14
4		7	9		*T*_10_	8		14	9	

**Table 4:** Discovered ML-HUIs from the database in Table 1.

ML-HUI	Utility	ML-HUI	Utility
Z	119	XD	95
ZY	277	XDC	108
Y	178	XC	140
XE	116	EDC	115
XED	93	EC	144
XEDC	119	DC	90
XEC	164	C	96

**Table 5:** Set of SML-HUIs.

SML-HUI	Utility
XEDC	119
XE	116
Z	119

The **MLHProtector** algorithm processes the the itemsets in the order of appearing. The order of processing the itemsets is *XEDC*,*XE* and *Z*.

For the itemset *XEDC*, it can be seen that the itemset is contain within the following transaction *T*_2_,*T*_3_ and *T*_4_. *S*_*g*_ = *XEDC*, *u*(*S*_*g*_) = *u*(*XEDC*) = 119. *diff*_*g*_ = 119 − 88 = 31. $DESC(S_{g},S)=ABEDC$. The algorithm is then processing each item in this set. Details are as follows.

- Item A:
  *sum*(*u*(*A*)) = ∑_*T*_*g*_ ∈ {*T*_2_, *T*_3_, *T*_4_}_*u*(*A*, *T*_*g*_) = 14 + 16 + 0 = 30.

$diff_{A}=\frac{sum(u(A))}{u(XEDC)}\times 31=\frac{30}{119}\times 31=7.815>0.$

  (*i*, *T*_*p*_) = *argmax*(*T*_*p*_ ∈ *T*(*S*_*g*_)) = *u*(*A*, *T*_3_) = 16 > *diff*_*A*_.
  Thus, (*iu*(*A*, *T*_3_)) is adjusted as follows:

$iu\left(A,T_{3}\right)=iu\left(A,T_{3}\right)-\lceil \frac{diff_{A}}{ptable_{A}}\rceil =8-\lceil \frac{7.815}{2}\rceil =4,diff_{A}=0.$

  Transaction *T*_3_ in D is then updated with *iu*(*A*, *T*_3_) = 4.
- Item B:
  *sum*(*u*(*B*)) = ∑_*T*_*g*_ ∈ {*T*_2_, *T*_3_, *T*_4_}_*u*(*B*, *T*_*g*_) = 0 + 3 + 4 = 7.

$diff_{B}=\frac{\text{sum}(u(B))}{u(XEDC)}\times 31=\frac{7}{119}\times 31=1.824>0.$

  (*i*, *T*_*p*_) = *argmax*(*T*_*p*_ ∈ *T*(*S*_*g*_)) = *u*(*B*, *T*_4_) = 4 > *diff*_*B*_.
  Thus, *iu*(*B*, *T*_4_) is adjusted as follows:

$iu\left(B,T_{4}\right)=iu\left(B,T_{4}\right)-\lceil \frac{diff_{B}}{ptable_{B}}\rceil =4-\lceil \frac{1.824}{1}\rceil =2$ , *diff*_*B*_ = 0.
  Transaction *T*_4_ in D then updated with *iu*(*B*, *T*_4_) = 2.
- Item E:
  *sum*(*u*(*E*)) = ∑_*T*_*g*_ ∈ {*T*_2_, *T*_3_, *T*_4_}_*u*(*E*, *T*_*g*_) = 10 + 18 + 14 = 42.

$diff_{E}=\frac{sum(u(E))}{u(XEDC)}\times 31=\frac{42}{119}\times 31=10.941>0.$

  (*i*, *T*_*p*_) = *argmax*(*T*_*p*_ ∈ *T*(*S*_*g*_)) = *u*(*E*, *T*_3_) = 9 > *diff*_*E*_.
  Thus, *iu*(*E*, *T*_3_) is adjusted as follows:

$iu\left(E,T_{3}\right)=iu\left(E,T_{3}\right)-\lceil \frac{diff_{E}}{ptable_{E}}\rceil =9-\lceil \frac{10.941}{2}\rceil =3$ , *diff*_*E*_ = 0.
  Transaction *T*_3_ in D is then updated with *iu*(*E*, *T*_3_) = 3.
- Item D:
  *sum*(*u*(*D*)) = 14. *diff*_*D*_ = 3.647 > 0.
  (*i*, *T*_*p*_) = *argmax*(*T*_*p*_ ∈ *T*(*S*_*g*_)) = *u*(*D*, *T*_3_) = 8 > *diff*_*D*_.
  Thus, *iu*(*D*, *T*_3_) is adjusted as follows:

$iu\left(D,T_{3}\right)=iu\left(D,T_{3}\right)-\lceil \frac{diff_{D}}{ptable_{D}}\rceil =8-\lceil \frac{3.647}{1}\rceil =4$ , *diff*_*D*_ = 0.
  Transaction *T*_3_ in D is then updated with *iu*(*D*, *T*_3_) = 4.
- Item C: *sum*(*u*(*C*)) = 26. *diff*_*C*_ = 6.773 > 0.
  (*i*, *T*_*p*_) = *argmax*(*T*_*p*_ ∈ *T*(*S*_*g*_)) = *u*(*C*, *T*_3_) = 12 > *diff*_*C*_.
  Thus, *iu*(*C*, *T*_3_) is adjusted as follows:

$iu\left(C,T_{3}\right)=iu\left(C,T_{3}\right)-\lceil \frac{diff_{C}}{ptable_{C}}\rceil =6-\lceil \frac{6.773}{2}\rceil =2$ , *diff*_*C*_ = 0.
  Transaction *T*_3_ in D is then updated with *iu*(*C*, *T*_3_) = 2.

After the itemset *XEDC* is processed, transaction *T*_3_ and *T*_4_ is sanitized as presented in Table 6

**Table 6:** Database after processed itemset *XEDC*.

Internal utility					TID	Utility				
A	B	C	D	E		A	B	C	D	E
4	3	2	4	3	*T*_3_	8	3	4	4	6
	2	5	3	7	*T*_4_		2	10	3	14

Similar operations are carried out on the itemsets *XE* and *Z*. The sanitized database D’ obtained after processing all given SML-HUIs using the **MLHProtector** algorithm is presented in Table 7. The affected transactions are *T*_2_,*T*_3_,*T*_4_,*T*_5_ and *T*_7_.

**Table 7:** Sanitized databases using MLHProtector algorithm.

TID	Items	Internal Utilities
*T*_1_	A, C	3, 7
*T*_2_	A, C, D, E	6, 2, 3, 5
*T*_3_	A, B, C, D, E	4, 3, 2, 4, 3
*T*_4_	B, C, D, E	2, 5, 3, 3
*T*_5_	A, D	9, 5
*T*_6_	A, C, E	3, 4, 4
*T*_7_	B, C, E	6, 7, 6
*T*_8_	C, D	2, 4
*T*_9_	C, D, E	8, 3, 7
*T*_10_	A, C, D	4, 7, 9

Using the same transformed database D in Table 3, and the taxonomy information provided in Fig 1. The same settings as previous example with *ξ* = 88 and the SML-HUIs in Table 5, the illustrative running of the **FMLHProtector** algorithm is described as follows.

The **FMLHProtector** sorts all the sensitive leaf nodes obtained using the descending order of their quantities per SML-HUI. Results are shown in Table 8.

**Table 8:** Sorted sensitive leaf nodes of SML-HUIs.

SML-HUI	Utility	Sorted sensitive leaf nodes
*XEDC*	119	*ABEDC*
*XE*	116	*ABE*
*Z*	119	*DE*

Similar to the **MLHProtector**, the **FMLHProtector** algorithm process the SML-HUIs in the order of appearance as in Table 8. The first itemset to be process is XEDC. **FMLHProtector** actually process all the sorted sensitive leaf nodes of *XEDC*, which is *ABEDC*.

Considering *S*_*L*_ = *ABEDC*, then *u*(*S*_*L*_) = *u*(*ABEDC*) = 119. *diff*_*L*_ = *diff*_*counter* = 119 − 88 = 31. Each sensitive leaf nodes in *S*_*L*_ is then scanned and processed in the respective order.

- Item A:
  *sum*(*u*(*A*)) = *u*(*A*, *T*_2_) + *u*(*A*, *T*_3_) + *u*(*A*, *T*_4_) = 30,

$diff_{A}=\frac{sum(u(A))}{u(ABEDC)}\times {\text{diff}}_{L}=7.815.$
  - *T*_*p*_ = *T*_2_: $rq=\lceil \frac{diff_{A}}{{\text{ptable}}_{A}}\times \frac{u(A,T_{2})}{sum(u(A))}\rceil =\lceil \frac{7.815}{2}\times \frac{14}{30}\rceil =2.$
    *iu*(*A*, *T*_2_) = *iu*(*A*, *T*_2_) − *rq* = 7 − 2 = 5. Update *diff*_*counter* = *diff*_*counter* − (*rq* × *ptable*_*A*_) = 31 − 4 = 27 ≥ 0.
  - *T*_*p*_ = *T*_3_: $rq=\lceil \frac{diff_{A}}{ptable_{A}}\times \frac{u(A,T_{3})}{sum(u(A))}\rceil =\lceil \frac{7.815}{2}\times \frac{16}{30}\rceil =3.$
    *iu*(*A*, *T*_3_) = *iu*(*A*, *T*_3_) − *rq* = 8 − 3 = 5.
    Update *diff*_*counter* = *diff*_*counter* − (*rq* × *ptable*_*A*_) = 27 − 6 = 21 ≥ 0.
  - *T*_*p*_ = *T*_4_: *iu*(*A*, *T*_4_) = 0.
- Item B: $sum\left(u\left(B\right)\right)=7,diff_{B}=\frac{sum(u(B))}{u(ABEDC)}\times diff_{L}=1.824.$
  - *T*_*p*_ = *T*_2_: *iu*(*B*, *T*_2_) = 0.
  - *T*_*p*_ = *T*_3_: $rq=\left\lceil \frac{diff_{B}}{ptable_{B}}\times \frac{u(B,T_{3})}{sum(u(B))}\right\rceil =\left\lceil \frac{1.824}{1}\times \frac{3}{7}\right\rceil =1$.
    *iu*(*B*, *T*_3_) = *iu*(*B*, *T*_3_) − *rq* = 3−1 = 2.
    Update *diff*_*counter* = *diff*_*counter* − (*rq* × *ptable*_*B*_) = 21 − 1 = 20 ≥ 0.
  - *T*_*p*_ = *T*_4_: $rq=\left\lceil \frac{diff_{B}}{ptable_{B}}\times \frac{u(B,T_{4})}{sum(u(B))}\right\rceil =\left\lceil \frac{1.824}{1}\times \frac{4}{7}\right\rceil =2$.
    *iu*(*B*, *T*_4_) = *iu*(*B*, *T*_4_) − *rq* = 4 − 2 = 2.
    Update *diff*_*counter* = *diff*_*counter* − (*rq* × *ptable*_*B*_) = 20 − 2 = 18 ≥ 0.
- Item E: *sum*(*u*(*E*)) = 42, $diff_{E}=\frac{sum(u(E))}{u(ABEDC)}\times diff_{L}=10.941.$
  - *T*_*p*_ = *T*_2_: *rq* = 2, *iu*(*E*, *T*_2_) = *iu*(*E*, *T*_2_) − *rq* = 5 − 2 = 3, *diff*_*counter* = 18 − 4 = 14 ≥ 0.
  - *T*_*p*_ = *T*_3_: *rq* = 3, *iu*(*E*, *T*_3_) = *iu*(*E*, *T*_3_)−*rq* = 9 − 3 = 6, *diff*_*counter* = 14−6 = 8 ≥ 0.
  - *T*_*p*_ = *T*_4_: *rq* = 2, *iu*(*E*, *T*_4_) = *iu*(*E*, *T*_4_)−*rq* = 7 − 2 = 5, *diff*_*counter* = 8−4 = 4 ≥ 0.
- Item D: *sum*(*u*(*D*)) = 14, *diff*_*D* = *sum*(*u*(*D*))/*u*(*ABEDC*) × *diff*_*L* = 3.647.
  - *T*_*p*_ = *T*_2_: *rq* = 2, *iu*(*D*, *T*_2_) = *iu*(*D*, *T*_2_) − *rq* = 7−2 = 5, *diff*_*counter* = 4−1 = 3 ≥ 0.
  - *T*_*p*_ = *T*_3_: *rq* = 3, *iu*(*D*, *T*_3_) = *iu*(*D*, *T*_3_) − *rq* = 8 − 3 = 5, *diff*_*counter* = 3−3 = 0 ≥ 0.
  - *T*_*p*_ = *T*_4_: *rq* = 1, *iu*(*D*, *T*_4_) = *iu*(*D*, *T*_4_) − *rq* = 3 − 1 = 2, *diff*_*counter* = 0 − 1 = −1 < 0.

At this iteration, since *diff*_*counter* is negative, the algorithm is done processing the itemset ABEDC since its utility has reduced to be lowered than the threshold *ξ* = 88, which is *u*(*ABEDC*) = 87. The affected transactions after processing *ABEDC* is presented in Table 9.

**Table 9:** Database after processed itemset *XEDC*.

Internal utility					TID	Utility				
A	B	C	D	E		A	B	C	D	E
5		2	2	3	*T*_2_	10		4	2	6
5	2	6	5	6	*T*_3_	10	2	12	5	12
	2	5	2	5	*T*_4_	0	2	10	2	10

Similarly, **FMLHProtector** continues to process *ABE* and *DE*. The sanitized database D’ after processing all three SML-HUIs are shown in Table 10.

**Table 10:** Sanitized database obtained from the FMLHProtector algorithm.

TID	Items	Internal Utilities
*T*_1_	A, C	3, 7
*T*_2_	A, C, D, E	4,2,1,2
*T*_3_	A, B, C, D, E	5,2,6,4,5
*T*_4_	B, C, D, E	2,5,1,5
*T*_5_	A, D	9, 7
*T*_6_	A, C, E	3, 4, 4
*T*_7_	B, C, E	6, 7, 6
*T*_8_	C, D	2, 3
*T*_9_	C, D, E	8, 2, 7
*T*_10_	A, C, D	4, 7, 7

## 5 Experimental evaluations

### Experimental setups

This section performs a series of experiments to evaluate the performance of **MLHProtector** and **FMLHProtector** with regard to hiding SML-HUIs. Six different databases were used in the experiments, as shown in Table 11. The databases used in this study is publicly available from the SPMF Open Source Data Mining Library. The characteristics of the databases are provided in Table 11.

**Table 11:** Characteristics of the databases used in the experiments.

Database	|D|	|I|	|G|	Taxonomy		Density(%)
				Type	Depth	
Chess	3,196	75	30	Synth.	3	49.33
Mushroom	8,124	119	26	Synth.	4	19.33
Fruithut	181,970	1,265	43	Real	4	0.28
Liquor	52,131	4,026	78	Real	7	0.20
Accidents	340183	468	225	Synth.	5	7.22
RecordLink	574,913	29	21	Synth.	6	38.48

As presented in Table 11, |D| denotes the size of each database in terms of number of transactions; |I| denotes number of specialized items in each database; |G| is the number of generalized items in its respective taxonomy; **Type** denotes the type of the taxonomy whether **synthetic** or **real**; **Depth** represents the number of levels in the taxonomy; *T*_*a*_*vg* and *T*_*m*_*ax* denotes the average transaction length and maximum transaction length, respectively; **Density** denotes the percentage of *T*_*a*_*vg* over |I|. These databases are obtained from the Open-Source Data Mining Library (SPMF) [54]. Except the Fruithut and Liquor databases which have the built-in taxonomy structures, Chess, Mushroom, Accidents and RecordLink have their taxonomy structure synthesized, which is included in the source code release of this work. Among the evaluated databases, RecordLink is the largest with over 500K transactions.

In all experiments, the *ξ* threshold is used as a relative value. Besides, the set of SML-HUIs is randomly selected from the MLHUIs discovered by the MLHUI-Miner algorithm. The percentage of SML-HUIs with regard to MLHUIs is denoted as SP. In the experiments, the values of *ξ* and *SP* are varied to evaluate the performance of the two algorithms **MLHProtector** and **FMLHProtector** with regard to hiding sensitive MLHUIs. In addition, the *HF*, *MC* and *AC* factors are also compared to evaluate the proposed algorithms.

The proposed algorithms were implemented in Java, using JDK8. All the experiments were conducted on a computer running Windows 10 Pro, equipped with an Intel® Core™ i5-6500, and has 8GB of memory.

Although the two proposed algorithms were extended from HHUIF and MSCIF [11], they are the firsts to address the task of hiding sensitive high utility itemsets from hierarchical databases, to the best of our knowledge. Operating in this type of database yields different outputs compared to the traditional transaction databases as the taxonomy structures provide the items that are not exist in the traditional databases. This enlarges the search space of the problem significantly. And thus, the runtime of both mining and sanitizing task are also much longer than those that operates on the database without the hierarchical information. Due to the reason, comparing runtime of the proposed algorithms against their original version might yield incorrect statistical data.

### Experimental results

#### The *HF* ratio

As presented in Definition 12, the *HF* factor denotes the ratio between the number of SML-HUIs that the sanitization process failed to hide over the number of selected SML-HUIs. In all four tests, the values are equal to zero for both **MLHProtector** and **FMLHProtector** as they successfully hide all the SML-HUIs in the set S from MLHUIM data miners. Thus, both algorithms have achieved the designated goal in PPUM.

#### The *MC* ratio

In these tests, the *MC*% factor denotes the ratio between the number of non-sensitive MLHUIs appearing in D but which cannot be discovered in D’ over the number the total number of non-sensitive MLHUIs in D. The results for evaluating the *MC* factor are shown in Tables 12 to 14. Table 12 presents the *MC*% value when keeping the *SP* threshold fixed and varying the *ξ* threshold, Table 13 is the comparisons when the *ξ* threshold is fixed and *SP* varied, and the comparison results on different abstract levels at fixed thresholds are shown at Table 14.

**Table 12:** *MC*% ratios when varying *ξ* and keeping *SP*% fixed.

Chess		Mushroom		Fruithut		Liquor		Accidents		RecordLink	
*SP* = 1.5		*SP* = 1.5		*SP* = 1.5		*SP* = 1.5		*SP* = 2.0		*SP* = 2.0	
*ξ*	MC	*ξ*	MC	*ξ*	MC	*ξ*	MC	*ξ*	MC	*ξ*	MC
**MLHProtector**											
36	100	7	99	0.10	82	0.05	62	34	98	5	95
40	100	9	99	0.15	73	0.10	53	36	100	8	94
44	100	11	99	0.20	62	0.15	48	38	95	11	69
46	100	13	98	0.25	60	0.20	41	40	98	14	75
48	99	15	87	0.30	52	0.25	25	42	97	17	85
**FMLHProtector**											
36	98	7	93	0.10	69	0.05	37	34	74	5	54
40	99	9	91	0.15	59	0.10	28	36	98	8	63
44	97	11	89	0.20	49	0.15	26	38	58	11	28
46	89	13	88	0.25	44	0.20	26	40	45	14	32
48	86	15	71	0.30	38	0.25	17	42	30	17	44

**Table 13:** *MC*% ratios when varying *SP*% and keeping *ξ* fixed.

Chess		Mushroom		Fruithut		Liquor		Accidents		RecordLink	
*ξ* = 40		*ξ* = 9		*ξ* = 0.15		*ξ* = 0.10		*ξ* = 44		*ξ* = 34	
*SP*	MC	*SP*	MC	*SP*	MC	*SP*	MC	*SP*	MC	*SP*	MC
**MLHProtector**											
1.0	100	1.0	100	1.0	66	1.0	39	2	98	2	95
1.2	100	1.2	100	1.2	70	1.2	45	3	100	3	94
1.4	100	1.4	99	1.4	70	1.4	49	4	95	4	69
1.6	100	1.6	99	1.6	72	1.6	68	5	98	5	75
1.8	100	1.8	99	1.8	78	1.8	71	6	97	6	85
**FMLHProtector**											
1.0	97	1.0	89	1.0	51	1.0	26	2	74	2	54
1.2	98	1.2	90	1.2	52	1.2	31	3	98	3	63
1.4	98	1.4	89	1.4	53	1.4	33	4	58	4	28
1.6	98	1.6	89	1.6	55	1.6	44	5	45	5	32
1.8	99	1.8	90	1.8	64	1.8	44	6	30	6	44

**Table 14:** *MC*% ratios at different abstraction levels *l*.

Chess		Mushroom		Fruithut		Liquor		Accidents		RecordLink	
*ξ* = 25		*ξ* = 3		*ξ* = 0.02		*ξ* = 0.01		*ξ* = 20		*ξ* = 0.01	
*SP* = 10		*SP* = 1		*SP* = 1		*SP* = 20		*SP* = 10		*SP* = 10	
*l*	*MC*	*l*	*MC*	*l*	*MC*	*l*	*MC*	*l*	*MC*	*l*	*MC*
**MLHProtector**											
1	100	1	100	1	1	1	7	1	100	1	100
2	100	2	100	2	79	2	50	2	100	2	99
3	18	3	31	3	95	3	60	3	100	3	37
		4	1	4	72	4	88	4	100	4	29
						5	91	5	95	5	15
						6	62			6	9
						7	10				
**FMLHProtector**											
1	82	1	84	1	1	1	6	1	100	1	54
2	100	2	81	2	71	2	66	2	99	2	49
3	10	3	17	3	90	3	62	3	95	3	18
		4	2	4	44	4	88	4	95	4	13
						5	72	5	14	5	7
						6	37			6	2
						7	4				

In Table 12 the returned result is the *MC*% factor of the two proposed algorithms on the four test databases when varying *ξ* and fixing *SP*. On dense databases such as Chess, Mushroom, Accidents and RecordLink, the obtained *MC*% factor is very close to 100%. This means almost all the non-sensitive MLHUIs are affected and were hidden along with the sensitive MLHUIs. When a SML-HUI is located from higher abstraction levels in a dense database, hiding this itemset could lead to a chain reaction on all transactions containting that itemset. The longer the itemsets, the stronger the side-effect on the database.

For the remaining two databases, Liquor and Fruithut, the *MC*% ratios are still higher than expected (50%). When an itemset is modified, the chance it impacts other non-sensitive MLHUIs is also higher, thus raising the side-effect ratio.

In Table 13, when varying SP and keeping *ξ* fixed, the *MC*% ratio rises as the *SP* ratio increases. This is due to the fact that when increasing the number of SML-HUIs, hiding them would cause greater side effects with regard to the non-sensitive MLHUIs.

Table 14 depicts the comparisons of the *MC*% ratio over all abstraction levels in the test databases. Considering the dense databases Chess, Mushroom, Accidents and RecordLink, both algorithms have a *MC*% ratio, almost 100%, at levels 1 and 2. SML-HUIs at these levels have a very high chance of appearing in many transactions, and the impact on non-sensitive MLHUIs is thus higher. Fortunately, synthetic databases such as the tested dense databases are not frequently occurred in real-world scenarios or applications. For the two sparse databases, Fruithut and Liquor, the *MC*% ratio is lower but most of the obtained results are higher than 50%.

As observed, in dense databases such as Chess, Mushrooms, Accidents and RecordLink, the *MC*% ratio of both algorithms is high, closing to 100%. This means almost non-sensitive MLHUIs are affected as the SML-HUIs are being hide. On the sparse databases Fruithut and Liquor, the ratio is lower, but it is still higher than expected (over 50%). Thus, performing data sanitizing on dense databases can cause more side-effect than on the sparse databases.

#### The *AC* ratio

Similar results as those for the *HF* factor is found for the *AC* factor. The *AC* factor denotes the number of MLHUIs in D’ but are not MLHUIs in D over the number of actual MLHUIs in D. Both **MLHProtector** and **FMLHProtector** return zeroes on all four test databases. Based on the desired goals, it can be seen that both algorithms do not produce any artificial MLHUIs in sanitized databases as they only reduce the items’ utilities.

#### Execution time

**Fig 3:**
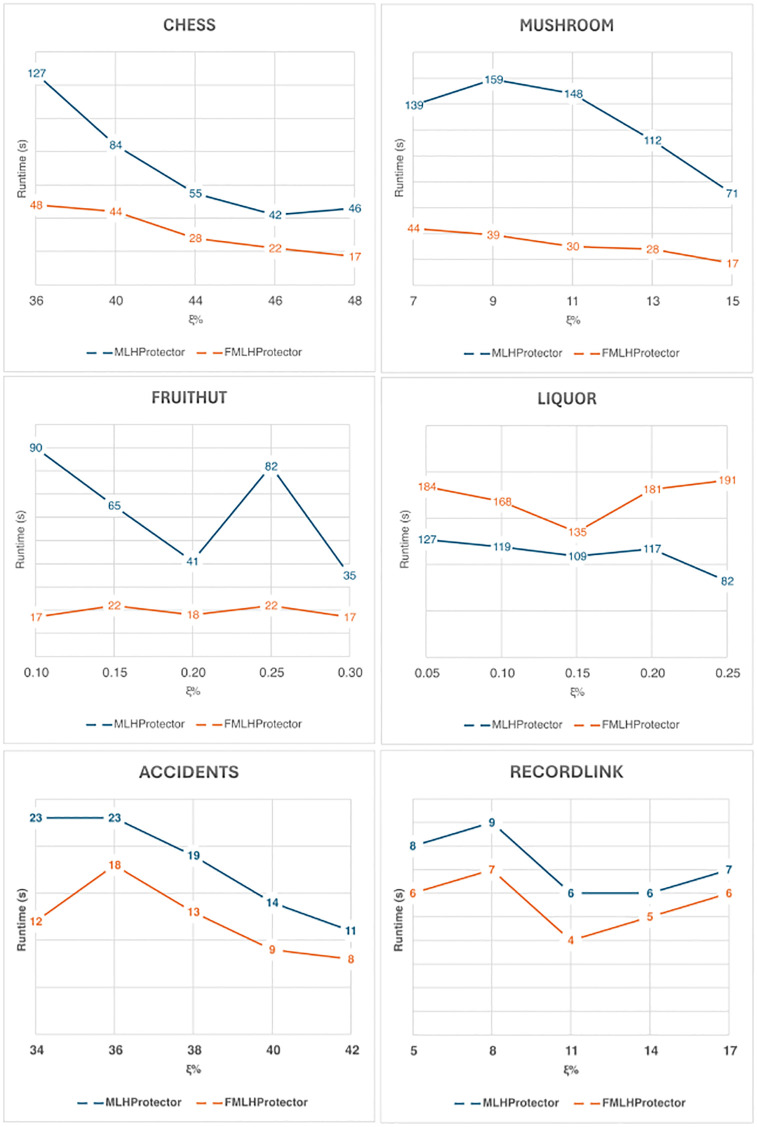
Runtime comparison when varying *ξ*.

**Fig 4:**
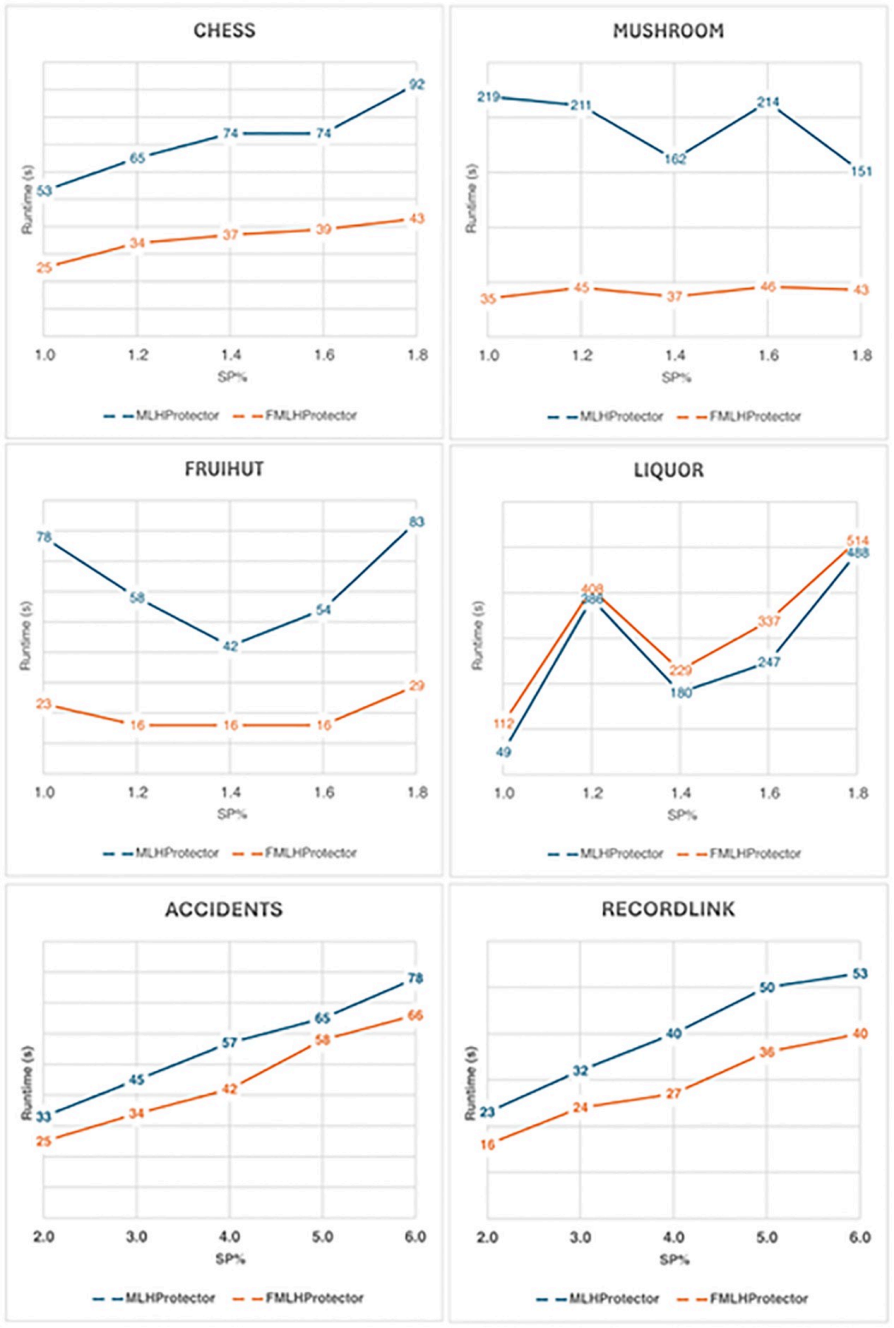
Runtime comparison when varying *SP*.

The runtime of the proposed algorithms, **MLHProtector** and **FMLHProtector**, are also obtained during the experiments, which were carried out multiple times. The values are averaged and visualized in Table 15 and Fig 3 when the threshold *ξ* is varied and *SP* is fixed at 1.50% on all databases; Table 16 and Fig 4 show the results when the *SP* is varied and *ξ* is locked at 40%, 9%, 0.15% and 0.10% for Chess, Mushroom, Fruithut and Liquor, respectively.

**Table 15:** Runtime of the proposed algorithms when varying *ξ*.

Chess		Mushroom		Fruithut		Liquor		Accidents		RecordLink	
*SP* = 1.5		*SP* = 1.5		*SP* = 1.5		*SP* = 1.5		*SP* = 2.0		*SP* = 2.0	
*ξ*	time (s)	*ξ*	time (s)	*ξ*	time (s)	*ξ*	time (s)	*ξ*	time (s)	*ξ*	time (s)
**MLHProtector**											
36	127	7	139	0.10	90	0.05	127	34	23	5	8
40	84	9	159	0.15	65	0.10	119	36	23	8	9
44	55	11	148	0.20	41	0.15	109	38	19	11	6
46	42	13	112	0.25	82	0.20	117	40	14	14	6
48	46	15	71	0.30	35	0.25	82	42	11	17	7
**FMLHProtector**											
36	48	7	44	0.10	17	0.05	184	34	12	5	6
40	44	9	39	0.15	22	0.10	168	36	18	8	7
44	28	11	30	0.20	18	0.15	135	38	13	11	4
46	22	13	28	0.25	22	0.20	181	40	9	14	5
48	17	15	17	0.30	17	0.25	191	42	8	17	6

**Table 16:** Runtime of the proposed algorithms when varying *SP*%.

Chess		Mushroom		Fruithut		Liquor		Accidents		RecordLink	
*ξ* = 40		*ξ* = 9		*ξ* = 0.15		*ξ* = 0.10		*ξ* = 44		*ξ* = 34	
*SP*	time(s)	*SP*	time(s)	*SP*	time(s)	*SP*	time(s)	*SP*	time(s)	*SP*	time(s)
**MLHProtector**											
1.0	79	53	1.0	70	219	1.0	66	78	1.0	69	49
1.2	94	65	1.2	84	211	1.2	79	58	1.2	82	386
1.4	110	74	1.4	98	162	1.4	92	42	1.4	96	180
1.6	126	74	1.6	112	214	1.6	105	54	1.6	109	247
1.8	141	92	1.8	126	151	1.8	119	83	1.8	123	488
**FMLHProtector**											
1.0	79	25	1.0	70	35	1.0	66	23	1.0	69	112
1.2	94	34	1.2	84	45	1.2	79	16	1.2	82	408
1.4	110	37	1.4	98	37	1.4	92	16	1.4	96	229
1.6	126	39	1.6	112	46	1.6	105	16	1.6	109	337
1.8	141	43	1.8	126	43	1.8	119	29	1.8	123	514

It can be observed that as the *ξ* lowered, the number of discovered ML-HUIs is increased. In the tests, the number of SML-HUIs in the set: S is also increased, which lead to the increased in sanitizing time. This is due to the time needed to process each SML-HUIs. Per SML-HUIs, each of its sensitive items are then processed by either adjusting/lowering the utility or removing them from respective transactions.

In most the databases, **MLHProtector** consumes more time finding sensitive items having maximum utility to process first, while **FMLHProtector** sorts them according to the descending order of the size of the itemsets before processing. Processing larger itemset first can transfer the effect to smaller ones, as the items which were already checked are skipped in subsequent iterations. This thus saves more runtime, especially on dense databases with long transactions. Overall, the runtimes of **FMLHProtector** in databases such as Chess, Mushrooms, Fruithut, Accidents and RecordLink are much better than that of **MLHProtector**, except for the database Liquor. This is because of the sparsity of the Liquor database, which is where the cost of sort operations become higher than the cost of SML-HUIs processing.

### Discussions

Based on the results of the experiments using both proposed algorithms, **MLHProtector** and **FMLHProtector**, we can observe the following.

- With the PPDM factors, such as *HF*, *MC* and *AC*, both algorithms have achieved the goal of hiding sensitive MLHUIs from hierarchical databases through utility reductions or item removals (*HF* = 0). The factor *AC* = 0 means the algorithms do not produce any artifical MLHUIs in the sanitized databases. However, both algorithms perform utility reduction on sensitive items. They thus cause utility-loss in the original database. This is the reason for the high *MC* ratios of both algorithms.
- In terms of execution time, **FMLHProtector** has better runtime than **MLHProtector** thanks to the optimizations employed. However, applying **FMLHProtector** on low density databases could lead to a slower runtime. Nonetheless, **FMLHProtector** still proves to be the most stable algorithm as it retains the original the database structure by keeping the sensitive items.

Based on the analysis, it can be seen that all the proposed algorithms cause side effects. The more SML-HUIs that need to be hidden, the higher the side effect’s impact (as observed from the MC% ratio). Considering the utility-loss side-effect when performing the sanitizing process could also help lower the impact.

## 6 Conclusions and future works

This work proposed the idea of hiding sensitive MLHUIs from hierarchical databases by extending the HHUIF algorithm. Two novel algorithms, named **MLHProtector** and **FMLHProtector**, were introduced to carry out this new task in the field of PPUM.

To achieve the goal, the algorithms adopt either the strategy of lowering an item’s utility or completely purging the item from the transactions. These strategies are leveraged to operate on hierarchical databases using the information provided by the database’s taxonomy.

Both proposed algorithms succeeded in hiding all sensitive MLHUIs from hierarchical databases using the mentioned strategies. To the best of our knowledge, they are the first works to address this new task in PPUM.

However, they still have the following limitations.

- Neither **MLHProtector** nor **FMLHProtector** consider the utility-loss side effect when hiding SML-HUIs.
- Furthermore, the impact of side effects with regard to non-sensitive information is also not considered by both algorithms. This cause the side-effect ratios are still high on the **MLHProtector**. For the **FMLHProtector**, the side-effects are much lower but on some tested databases, the algorithm still suffer from high missing costs.
- Besides, **MLHProtector** and **FMLHProtector** are not scalable when applied to large databases. It can be seen that the runtime of **MLHProtected** algorithm on several tested databases are very high. Although the **FMLHProtector** algorithm employs a strategy to reduce the sanitization time, it still has high runtime on large or sparse databases such as Liquor, Accidents and RecordLink.

The above-mentioned drawbacks are worth considering in future studies of this new mining task to improve the privacy-preserving performance. Besides, it could be applying extending the privacy-preserving mining task to other extensions of the high-utility mining, such as high-occupancy mining, high-average utility mining, etc.
